# The Desire for Amputation or Paralyzation: Evidence for Structural Brain Anomalies in Body Integrity Identity Disorder (BIID)

**DOI:** 10.1371/journal.pone.0165789

**Published:** 2016-11-10

**Authors:** Rianne M. Blom, Guido A. van Wingen, Sija J. van der Wal, Judy Luigjes, Milenna T. van Dijk, H. Steven Scholte, Damiaan Denys

**Affiliations:** 1 Department of Psychiatry, Academic Medical Center, University of Amsterdam, Amsterdam, The Netherlands; 2 Brain Imaging Center, Academic Medical Center, University of Amsterdam, Amsterdam, The Netherlands; 3 Department of Psychology, University of Amsterdam, Amsterdam, The Netherlands; 4 Netherlands Institute for Neuroscience, an institute of the Royal Netherlands Academy of Arts and Sciences, Amsterdam, The Netherlands; 5 Sackler Institute of Graduate Biomedical Sciences, New York University School of Medicine, New York, NY, United States of America; University of Zurich, SWITZERLAND

## Abstract

**Background:**

Body Integrity Identity Disorder (BIID) is a condition in which individuals perceive a mismatch between their internal body scheme and physical body shape, resulting in an absolute desire to be either amputated or paralyzed. The condition is hypothesized to be of congenital nature, but evidence for a neuro-anatomical basis is sparse.

**Methods:**

We collected T1-weighted structural magnetic resonance imaging scans on a 3T scanner in eight individuals with BIID and 24 matched healthy controls, and analyzed the data using voxel-based morphometry.

**Results:**

The results showed reduced grey matter volume in the left dorsal and ventral premotor cortices and larger grey matter volume in the cerebellum (lobule VIIa) in individuals with BIID compared to controls.

**Conclusion:**

The premotor cortex and cerebellum are thought to be crucial for the experience of body-ownership and the integration of multisensory information. Our results suggest that BIID is associated with structural brain anomalies and might result from a dysfunction in the integration of multisensory information, leading to the feeling of disunity between the mental and physical body shape.

## Introduction

We are supposed to have an internal body scheme that can distinguish our physical body from other objects [1,2]. However, there are cases in which persons feel that a specific body part is superfluous or alien. Such is the case in stroke patients who have suffered a hemorrhage in the parietal cortex and request the nurse to remove ‘that strange leg’ from their bed [3–5]. Individuals with Body Integrity Identity Disorder (BIID) experience a difference between their internal body scheme and physical body shape from early youth on. As a result, they desire to be either amputated or paralyzed, in order to neutralize this conflicting internal and external body (scheme) [6–8]. Despite clear observed disturbance in brain structures in body-image stroke patients [3–5,9], there are few studies that can provide evidence for brain abnormalities in BIID [10,11].

Originally BIID was seen as a mere psychological problem [12–14]. The obsession to amputate one’s limbs was called apotemnophilia and seen as an erotic manifestation of hatred towards maternal figures [12,13]. Later on, the incongruence between one’s experienced and assigned body was observed [15,16]. Parallels -the ‘not feeling well in one’s body’- were made with Gender Dysphoria, and the condition was classified as an identity disorder (BIID) [7]. Nowadays, the disorder is hypothesized to be a congenital cerebral disorder [17–21]. The early onset, the therapeutically unchangeable desire, the exact boundaries of the modification, and parallels in the symptomatology of several neurological conditions with an abnormal bodily experience (e.g. somatoparaphrenia or misoplegia) strengthen this hypothesis [14].

Next to the wish for limb amputation a milder variant of BIID is reported as well: the desire for non-functioning limbs [6,8,16]. The so-called paralyzation variant is characterized by the same typical BIID features [6,8,16,22], with the exception of a higher percentage of females [6,8]. Important to note, individuals with paralyzation BIID, only aim for non-functioning of the limbs and they prefer to keep the functions of the urogenital tract. In general, individuals with BIID do not aim for disability in order to obtain any sort of benefit other than feeling at ease with their own body. There is no conversion, imagined ugliness, pain or psychosis that could explain the desirability of body-modification [6]. Due to embarrassment for their bizarre wish, subjects with BIID rarely present themselves to medical professionals. This complicates investigating the disorder properly and results in the disorder not being officially recognized by the WHO [6,7].

Models of body-ownership are mainly based on studies with healthy individuals and patients with brain lesions. Studies in healthy individuals have often used an experimental paradigm called the rubber hand illusion (RHI). In RHI a fake hand will be perceived as one’s own when the actual hand is touched simultaneously with the fake one and only the latter is seen [23]. It is hypothesized that this illusion comes about by integration of multisensory (visual, proprioceptive and tactile) stimuli and a pre-existing body scheme that contains a reference description of the body [1,2]. In general, important brain areas involved in this illusion are the visual and primary sensory (SI) cortices that are involved in recognizing multisensory stimuli, the parietal cortex that is involved in multimodal integration [1,2,24]. Furthermore, most RHI studies report that the ventral premotor cortex is crucial to the emergence of the ownership experience [1,24–28], although there is some evidence that the insular cortex is also involved in the maintenance of the sense of body ownership [2,29].

Lesions in brain areas involved in this body ownership network lead to a variety of neurological body-ownership pathologies [4,30]. Somatoparaphrenia patients misidentify the ownership of a left-sided body part [3,30]; in misoplegia the loss of recognition goes along with hatred of a body part [31,32]; in alien hand syndrome involuntary movements and estrangement from a particular limb are prominent [33,34] and asomatognosonia patients lack awareness of a limb [30], although sometimes this misconception can (temporarily) be corrected. The right posterior insula seems to be a crucial structure in patients with disturbed sensation of limb ownership [4,9], but some case-studies also report patients with alien hand syndrome and asomatognosia with single lesions in the corpus callosum [33,35] or premotor cortex [36] respectively.

One study investigated structural anomalies in subjects with BIID. In 2013 Hilti et al preformed structural imaging in 13 male patients with a strong desire for amputation and an equal number matched controls [10]. Surface-based morphometry revealed group differences in the somatosensory and parietal cortices, although the results did not remain significant after correcting for multiple comparisons. Hilti et al. assumed that BIID is not a disorder that can be localized in any circumscribed region of the human brain, but assumed that it reflected a breakdown of key areas coding for different facets of the experience of body-ownership.

We recently reported the results of a functional MRI study in individuals with BIID [11]. We included 5 BIID patients with a single leg amputation desire and matched them to healthy controls. Our study showed that individuals with BIID have overall heightened responsivity of a large somatosensory network during tactile stimulation of both legs. Importantly, we found reduced activity in the left premotor cortex during stimulation of the disowned leg compared to the owned leg, suggesting that altered somatosensory processing in the premotor cortex is associated with the feeling of disownership in BIID.

To investigate whether altered processing in the premotor cortex is associated with differences in neuroanatomy, we now also analyzed the structural MRI data from our previous study in a slightly larger sample of participants. In line with our previous findings, we hypothesized that BIID could be associated with differences in cortical gray or white matter volume of the premotor cortices.

## Methods

### Participants

BIID participants were recruited through advertisements on online BIID forums and via other participants and were matched to healthy controls (HC). In order to increase power we collected thrice as many HC as individuals with BIID. BIID was diagnosed with a psychiatric assessment, using the definition of BIID as the existence of a lifelong desire to have either an amputation or paralyzation with the primary objective to restore one's true identity. Furthermore, patients were screened for the presence of DSM-IV axis I disorders. The study was approved by the Medical Ethical committee of the Academic Medical Centre (AMC) of the University of Amsterdam and written informed consent was obtained after complete description of the study to the subjects.

### Image acquisition

We acquired a T1-weighted whole brain high-resolution MRI on a 3 Tesla Intera full-body scanner (Philips Healthcare, Best, The Netherlands), equipped with a SENSE eight-channel receiver head coil, with the following parameters: matrix size: 256 × 256, field of view (FOV): 226 × 226 × 218 mm^3^, voxel size: 0.88 × 0.88 × 1.2 mm^3^, repetition time (TR): 9.6 msec., echo time (TE): 4.6 msec.; Flip angle: 8°; no. slices: 182. Head movement was restricted using foam padding.

### Image processing

We examined images for scanning and motion artefacts and large anatomical abnormalities before processing. Subsequently, we centered all images manually at the anterior commissure and pre-processed them with SPM8 (https://www.fil.ion.ucl.ac.uk/spm/sofware/) implemented in Matlab 8.1.0 (The Mathworks Inc., Natick, MA). Pre-processing for VBM included segmenting structural T1 images into grey matter (GM), white matter (WM), cerebrospinal fluid and skull, manually quality control check, normalization to MNI space, modulation with the Jacobian determinant, and spatial smoothing (Gaussian kernel of 12-mm full-width at half maximum), using the standard tools of SPM8. The obtained images were used for statistical analysis.

### Statistical analyses

We compared demographics using non-parametric tests (the Mann-Whitney *U* test) for linear variables and chi-square test for dichotomous variables in SPSS (IBM SPSS for Windows, 20.0, 2011). Voxel-wise statistical tests were family-wise error (FWE) rate corrected for multiple comparisons (p<0.05) across the whole brain or the region of interest (ROI), and were corrected for total intracranial volume (TIV) by including TIV as covariate of no interest. Given our a-priori knowledge of the involvement of the left dorsal and ventral premotor cortex related to BIID in our previous study, we applied a small volume correction (SVC) to these regions by centring a sphere of 15mm radius around the meta-analytic center of the premotor cortices based on a study by Mayka et. al. (2006) (i.e. PMd x = -20; y = -4; z = 58 and PMv x = -50; y = 5; z = 22) [37]. Moreover, for exploratory purposes we applied SVC to regions found in the only other BIID MRI paper of Hilti et al. by centring spheres of 15mm radius around their peak-coordinates found (i.e. superior parietal lobule x = 17 y = -50 z = 61; inferior parietal lobule x = 57 y = -27 z = 38; central sulcus x = 33 y = -16 z = 40; anterior insular cortex, upper cluster y = 32 x = 25 z = 9; anterior insular cortex, lower cluster x = 32 y = 20 z = -4; primary somatosensory cortex x = 5 y = -38 z = 62; secondary somatosensory cortex x = 54 y -3 z = 9; inferior parietal lobule x = 35 y = -31 z = 42) and combined those into one region of interest. In addition we reported clusters of more than 10 contiguous voxels, tresholded at p<0.005 uncorrected for multiple comparisons.

## Results

### Subjects

We recruited eight individuals with BIID and 24 matched healthy controls without a neurological or psychiatric history (Table 1). Characteristics of subjects with BIID are described in Table 2. In summary, two of the BIID participants had a psychiatric co-morbid mood disorder, resulting from having to deal with BIID, a strange, untreatable condition. Three individuals with BIID desired amputation of the right leg, two the left leg, one desired both legs to be amputated and two desired both legs to be paralyzed.

**Table 1:** Subject characteristics and global brain measures of BIID patients (BIID) and healthy controls (HC).

	BIID n = 8	HC n = 24
**Males (n–(%))**	7/8 (87.5)	21/24 (87.5)
**Age in years (mean–(sd))**	39 (10.2)	41 (10.6)
**High education level**^a^ **(n–(%))**	6/8 (75)	14/17 (82.4)^b^
**Right handed (n–(%))**	6/8 (75)	15/17 (92.2)^c^
**White matter volume cm**^**2**^ **(mean (sd))** ^d^	561.50 (42.6)	541.29 (53.1)
**Gray matter volume cm**^**2**^ **(mean (sd))** ^d^	648.00 (52.5)	632.67 (65.0)
**Total intracranial volume cm**^**2**^ **(mean (sd))** ^d^	1596.62 (105.9)	1572.25 (138.37)

^a^ High education level = > 16 years of education.

^b^ Missing variables for 7 subjects;

^c^ Missing variables for 7 subjects.

^d^MWU-test, p = ns

**Table 2:** Subject characteristics of Body Integrity Identity Disorder (BIID) patients.

Subject	Sex	Age (years)	Educational level^a^	Handedness	Desired modification^b^	Onset BIID (years)	Psychiatric history
**1**	Female	31	High	R	Both legs paralysis	5	Dysthymia
**2**	Male	43	High	R	L upper leg amputation	7	None
**3**	Male	47	High	R	Both upper legs amputation	8	None
**4**	Male	33	High	L	R upper leg amputation	5	None
**5**	Male	46	High	R	L upper leg amputation	4	None
**6**	Male	45	Low	R	R upper leg amputation	8	Depressive episode
**7**	Male	47	High	R	Lower back paralysis	10	None
**8**	Male	19	Low	L	R upper leg amputation	7	None

^a^ Low = ≤ 16 years of education; High = >16 years of education

^b^ L = left, R = right

### Voxel-based morphometry data

**Fig 1:**
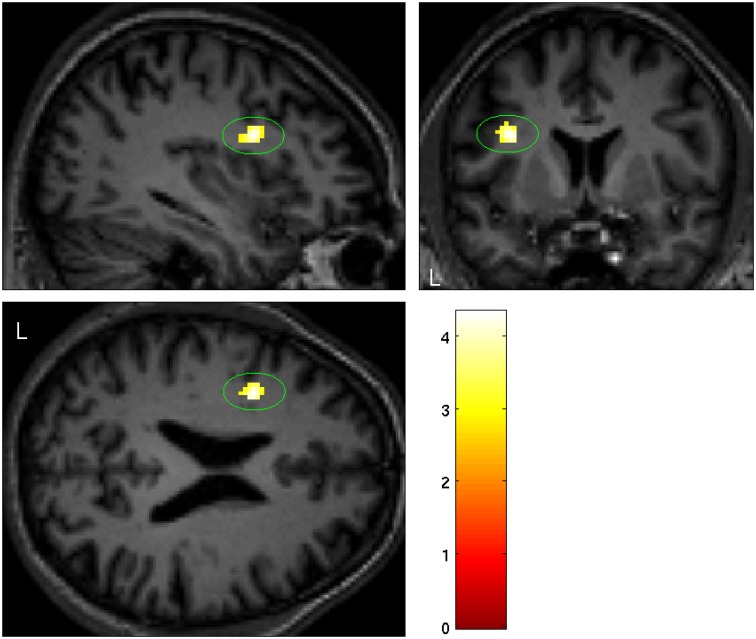
Significant smaller grey matter volume in the left ventral premotor cortex in BIID subjects (PMv: p = 0.006 (SVC), T = 4.27,maximum difference at MNI x = -36, y = 6, z = 22).

**Fig 2:**
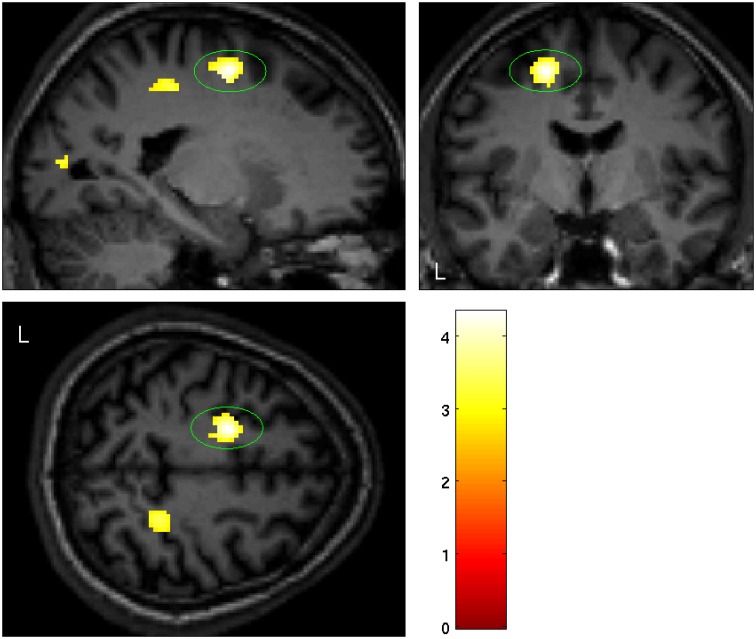
Significant smaller grey matter volume in the left dorsal premotor cortex in BIID subjects (PMd: p = 0.007 (SVC), T = 4.33, maximum difference at MNI x = -18; y = -6; z = 52).

**Fig 3:**
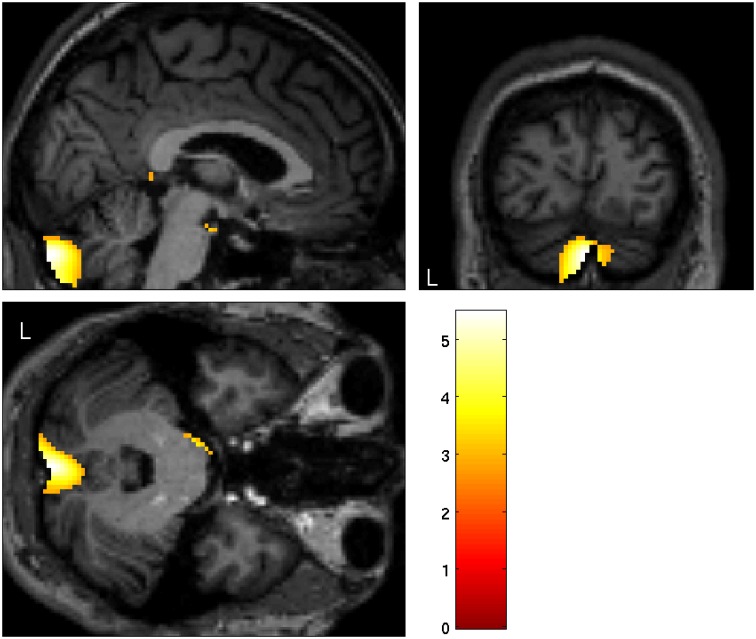
Significant larger grey matter volume in the left cerebellum in BIID subjects (p = 0.038 FWE corrected), T = 5.47, maximum difference at MNI x = -2, y = -90, z = -34).

Global brain measures did not differ significantly between groups (Table 1). Gray matter volumes in both the left PMd and left PMv were significantly smaller in BIID patients than in healthy controls (Figs 1 and 2). Gray matter volume of the left cerebellum (lobule VII) was significantly larger in BIID patients than in healthy controls (Table 3, Fig 3). To explore whether there the BIID patients in our study showed similar gray matter abnormalities as reported in the only other study on BIID, we created an ROI that included all the regions that were reported in Hilti et al. The brain region with the maximum difference was located in at the right inferior parietal lobule, but did not survive the SVC (p = 0.191 (SVC), T = 3.54, maximum difference at MNI x = 24 Y; y = -36; z = 48). There were no other significant differences in gray or white matter after correction for multiple comparisons. For exploratory purpose, results at p<0.005 uncorrected were reported in Tables 3 & 4.

**Table 3:** Results from the whole brain analysis corrected for total intracranial volume for *grey* matter volume differences between BIID patients (BIID) and healthy controls (HC). Values are displayed for clusters with >10 voxels at an initial threshold of p <0.005 uncorrected; L: left; R: right.

Anatomical region	L/R	MNI coordinates			Cluster size	T	Z	Peak level P(FWE-corr)	Peak level P(uncorr)	Larger volume in
		x	y	z						
**Ventral premotor cortex (PMv)**	L	-36	6	22	66	4.33	3.77	0.006^a^	<0.001	HC
**Dorsal premotor cortex (PMd)**	L	-18	-6	52	166	4.27	3.73	0.007^a^	<0.001	HC
**Sub-gyral (temporal lobe)**	R	44	-16	-20	116	3.58	3.23	0.888	0.001	HC
**Sub-gyral (parietal lobe)**	R	24	-38	50	120	3.55	3.21	0.900	0.001	HC
**Cuneus**	L	-22	-82	10	60	3.54	3.20	0.906	0.001	HC
**Parahippocampale gyrus**	R	18	-16	-20	13	3.49	3.16	0.925	0.001	HC
**Sub-gyral (parietal lobe)**	L	-20	-34	46	36	3.31	3.02	0.973	0.001	HC
**Supramarginal gyrus (parietal lobe)**	L	-44	-42	30	41	3.24	2.97	0.983	0.002	HC
**Superior temporal gyrus (temporal lobe)**	L	-40	-36	6	16	3.06	2.82	0.996	0.002	HC
**Cerebellum (Lobule VIIa, Crus II)**	L	-2	-90	-34	902	5.47	4.50	0.038	<0.001	BIID
**Pons**	L	-12	-22	-26	388	4.43	3.84	0.320	<0.001	BIID
**Superior medial frontal gyrus**	R	12	62	34	10	3.21	2.94	0.986	0.002	BIID
**Anterior cingulate cortex**	R	2	-40	2	32	3.14	2.89	0.992	0.002	BIID
**Parahippocampale gyrus**	R	34	-48	-8	53	3.17	2.91	0.990	0.002	BIID
**Cerebellum (Lobule NA)**	R	38	-70	-60	25	2.96	2.75	0.998	0.003	BIID

^a^ P-value after small volume correction

**Table 4:** Results from the whole brain analysis corrected for total intracranial volume for *white* matter volume differences between BIID patients (BIID) and healthy controls (HC). Values are displayed for clusters with >10 voxels at an initial threshold of p <0.005 uncorrected; L: left; R: right.

Anatomical region	L/R	MNI coordinates			Cluster size	T	Z	Peak level P(FWE-corr)	Peak level P (uncorr)	Larger volume in
		x	y	z						
**Superior Frontal Gyrus**	L	-14	26	58	18	3.47	3.15	0.923	0.001	HC
**Superior Temporal Gyrus**	R	58	-12	-6	46	3.36	3.06	0.923	0.001	HC
**Sub-gyral (frontal lobe)**	L	-20	-6	54	90	4.16	3.65	0.478	<0.001	BIID
**Inferior Frontal Gyrus**	L	-44	10	26	36	3.95	3.50	0.633	<0.001	BIID
**Middle Frontal Gyrus**	L	-24	54	14	88	3.92	3.48	0.654	<0.001	BIID
**Cerebellum posterior lobe**	L	-14	-68	-38	398	3.63	3.27	0.847	0.001	BIID
**Supramarginal gyrus (parietal lobe)**	R	42	-50	28	36	3.33	3.04	0.964	0.001	BIID
**Inferior Parietal Lobe**	L	-52	-42	28	21	3.19	2.93	0.986	0.002	BIID
**Cerebellum anterior lobe**	R	18	-60	-32	143	3.07	2.83	0.995	0.002	BIID
**Cingulate gyrus**	R	6	-20	28	25	2.99	2.77	0.998	0.003	BIID
**Thalamus**	R	16	-10	14	15	2.90	2.70	0.999	0.004	BIID

## Discussion

In this study we investigated structural brain differences in 8 BIID subjects with either a paralyzation or amputation variant using VBM. As expected we found significantly reduced grey matter volume in the left dorsal (PMd) and ventral (PMv) premotor cortices in BIID subjects compared to controls. Moreover, we additionally observed larger grey matter volume in the cerebellum (lobule VIIa) of BIID subjects compared to HC participants.

In line with the altered PM processing that we reported earlier in our functional MRI study in BIID individuals, we found structural PM gray matter differences [38]. It is accepted that the integration of visual, tactile and proprioceptor information across different body parts contributes to the perception of body ownership [1,2,39,40]. Activity in the premotor cortex (PM) has been shown to reflect the feeling of ownership of a limb by cortical integration of this multisensory information [27,38,39]. Especially the PMv is hypothesized to be crucial in the self-attribution of body-parts. The PMv is anatomically connected to somatosensory and visual areas in the posterior parietal cortex and to frontal motor areas [39,41].

In perceptual illusions and our BIID study the PM seems crucial in experiencing body-ownership, while in stroke patients with disturbance in body-image (n = 70), no difference in PM connectivity was found [25]. Body-ownership problems in neurological patients are mainly seen as impairment of the parietal cortex and insula [9,42]. From a clinical point of view, the loss of recognition of a particular body part is prominent in those neurological patients [3,30], whereas subjects with BIID admit that their limbs are theirs but experience an internal struggle of feeling overcomplete with four functioning limbs [6,14,18]. Therefore, PMv could be more related to the higher order inner body-experiences, whereas the parietal and insular cortex deficits result in a more primary disturbance when it comes to processing visual and sensory information leading to recognition of body-parts. This is underlined by a recent study of Lenggenhager et al. in which 9 BIID subjects were able to experience a rubber foot illusion, although the vividness of the illusion was stronger than in controls [43]. Following this hypothesis, the paralyzation-variant can be seen as a ‘milder form of BIID’, disintegrating the multisensory information to the extent that limbs are not functioning. Whereas a stronger dysfunction might lead to stronger desire, i.e. amputation of the limbs.

One can only speculate about the possible mechanisms underlying the gray matter differences in the PM in BIID, as differences in gray matter could lead to or may be the consequence of abnormal body experience and PM processing. BIID subjects report symptoms of estrangement from early youth on [6,7]. This strengthens the idea of a congenital cause of the condition [17]. The gray matter reductions might therefore be attributed to an early developmental problem. On the other hand, reduced neuronal activity in the PM could lead to structural changes of this area.

The finding of enlarged gray volume in the cerebellum (lobule VIIa, crus II) was unexpected. From an anatomical point of view prefrontal and posterior parietal cortices are reciprocally interconnected with cerebellar lobule VIIa [44]. Functionally this lobule is involved in emotional and cognitive processing [45].

In another psychiatric body-image disorder structural cerebellar differences have been found. In anorexia nervosa (AN) patients, in which a distorted body image is a major component of their condition, atrophy of crus I and II of the cerebellum was found and related to mood alterations and cognitive rigidity [46,47]. However, a meta-analysis of structural MRI data in AN patients did not confirm this cerebellar volumetric differences [48]. Little is known about emotional and cognitive processing in BIID. Clinical studies in BIID report mood symptoms accompanying an unsolved desire [6,22]. Remarkably those depressive symptoms disappear after (self) amputation of their limb [20,49,50]. Through limbic projections on the cerebellum, this structure is an important region in neuroanatomical models of mood-disorders [51]. One study formally assessed emotional processing in BIID [52]. Seven individuals with BIID were tested in facial emotion recognition and emotional responses to disgusting images and, in general, showed no emotional impairment [52]. Functional imaging data are needed to test whether over-activity of the emotional brain circuitry, including the cerebellum, is present in BIID as a result of life-long depressive symptoms.

In addition to emotional processing, the cerebellum is also suggested to be involved in multisensory integration leading to the feeling of body-ownership, next to the vPM. Cerebellar activity, as well as premotor activity, was found to correlate with the strength of the ownership illusion in healthy controls [24,39]. Whereas in patients with neurodegenerative cerebellar ataxia, a reduced sense of the subjective illusory experience was observed [53]. Strikingly enough, the PM was intact in those patients, suggesting that an intact ventral premotor cortex is not sufficient to generate an intact illusion [53]. Moreover, our fMRI study showed that the cerebellum displays heightened activity in BIID during tactile stimulation [38]. Overall, one could hypothesize that, in BIID subjects, impaired PM functioning (reduced GM) leads to over-activity of the lobule VIIa of the cerebellum, leading, in turn, to increased volumetric differences of this structure.

One other neuro-imaging study investigated structural brain abnormalities in BIID. It reported subtle differences in cortical thickness and surfaces area across the insula and parietal cortex, although those results did not survive correction for multiple comparisons [19]. Differences in the PM and cerebellum were not reported but were also not included as regions of interest. Furthermore, our results show also differences in the parietal cortex at an exploratory statistical level and partly replicate the results of Hilti et al. In both studies structural gray matter abnormalities in the superior and inferior parietal lobe and frontal lobe were found. These might be the result of altered neuronal activity in the frontoparietal network involved in the experience of body-ownership in BIID [17,38,54]. The finding of structural frontoparietal abnormalities in two BIID studies provides evidence for the actual presence of these deviations, and therefore point to the direction that the anomalies may not reach statistical significance as a result of limited statistical power due to a limited number of these rare cases included in both studies.

This is the first VBM study reported in BIID subjects, including both the paralyzation and amputation variant of BIID, assuming both are of the same disorder since restoring the primary body identity is the main motivation for body modification, and both disorders only included disunity of the limbs (i.e. not the urogenital tract). After the main analysis, we would have preferred to stratify on both variants, although the number of subjects was not sufficient enough to do so. In addition, the small sample places limitations on generalizability. However, a sample size of eight affected subjects is still considered reasonable due to the rarity of BIID. To increase statistical power, we included three times as many healthy controls.

In conclusion, we found volumetric differences in BIID in the left premotor cortex and cerebellum (left lobule VII) using VBM. Both structures are hypothesized to be crucial for the experience of body-ownership and to integrate multisensory information. Considering these findings BIID could result from a dysfunction in the integration of multisensory information causing the feeling of disunity between the mental and physical bodyshape. Further research is warranted to replicate these findings and correlate them to fMRI data. More importantly, this evidence underlines the suggestion of a neural basis of BIID, and turns away from classifying the symptoms as a mere psychological condition. This is particularly important for the acknowledgment of the disorder as official in ICD-11, which will facilitate the quest to targets for new therapies.

## Supporting Information

S1 Dataset Raw clinical data. (SAV)

S2 Dataset Raw volumetric data. (XLSX)
